# Deep Link-Prediction Based on the Local Structure of Bipartite Networks

**DOI:** 10.3390/e24050610

**Published:** 2022-04-27

**Authors:** Hehe Lv, Bofeng Zhang, Shengxiang Hu, Zhikang Xu

**Affiliations:** 1 School of Computer Engineering and Science, Shanghai University, Shanghai 200444, China; hhlv@shu.edu.cn (H.L.); shengxianghu@shu.edu.cn (S.H.); xuzhikangnba@shu.edu.cn (Z.X.); 2 School of Computer and Information Engineering, Shanghai Polytechnic University, Shanghai 201209, China

**Keywords:** link prediction, bipartite network, local structure, representation learning

## Abstract

Link prediction based on bipartite networks can not only mine hidden relationships between different types of nodes, but also reveal the inherent law of network evolution. Existing bipartite network link prediction is mainly based on the global structure that cannot analyze the role of the local structure in link prediction. To tackle this problem, this paper proposes a deep link-prediction (DLP) method by leveraging the local structure of bipartite networks. The method first extracts the local structure between target nodes and observes structural information between nodes from a local perspective. Then, representation learning of the local structure is performed on the basis of the graph neural network to extract latent features between target nodes. Lastly, a deep-link prediction model is trained on the basis of latent features between target nodes to achieve link prediction. Experimental results on five datasets showed that DLP achieved significant improvement over existing state-of-the-art link prediction methods. In addition, this paper analyzes the relationship between local structure and link prediction, confirming the effectiveness of a local structure in link prediction.

## 1. Introduction

A bipartite network contains two types of nodes, and there are edges between different types of nodes [1]. Link prediction based on bipartite networks aims to predict the possibility of a link between two different types of nodes in the network [2,3]. Bipartite networks are ubiquitous, such as user–item purchase networks, drug–disease treatment networks, and author–subject research networks [4,5,6]. Link prediction based on bipartite networks can not only mine the hidden edge relationship between different types of nodes to provide the basis for network application, but also reveal the inherent law of network evolution to realize the dynamic analysis of the network.

**Figure 1:**
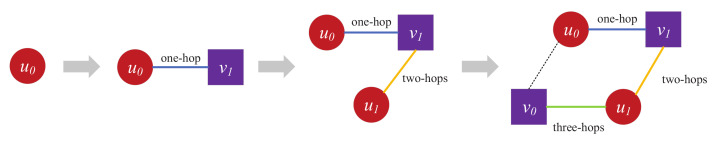
Link relationship between $u_{0}$ and $v_{0}$ in a bipartite network, where dashed lines represent possible edges.

Traditional link prediction is mainly based on the structure of the homogeneous network for target nodes. For example, common neighbor (CN) and preferential attachment (PA) mainly perform link prediction on the basis of common neighbors of target nodes by only involving the single-hop neighbors of target nodes [7]. Adamic–Adar (AA) and resource allocation (RA) are based on common neighbors of the neighbors of the target nodes as the link prediction basis, mainly based on two-hop neighbors of target nodes [8]. Katz is based on all paths connected by target nodes, involving multihop neighbors between target nodes [9]. Different from homogeneous networks, the neighbor of node *u* in a bipartite network is always node *v*, and the neighbor of node *v* is always node *u*, where *u* and *v* denote two types of nodes. For the structural characteristics of a bipartite network, similar relationships between neighbors still exist. For example, in a bipartite network, edges only exist between nodes of different types, and there are no edges between nodes of the same type. As shown in Figure 1, for different types of nodes $u_{0}$ and $v_{0}$, node $u_{0}$ reaches node $v_{0}$ through three hops, which is the shortest path between different types of nodes. Therefore, three-hop paths between node $u_{0}$ and $v_{0}$ can be used as indicators for link prediction. For example, by modeling drug–protein bipartite networks in biological networks, and on the basis of connectivity paths between target nodes, interactions between drugs and target proteins can be effectively achieved [10]. The connection of user–item–user–item in the recommender system is a recommendation indicator. The essence of recommendation is to predict the relationship between users and items, which is actually a variant of link prediction [11,12]. These link predictions are mainly based on the connectivity paths between target nodes in which network structure information is extracted. On the basis of the length of the connected paths between target nodes, extracted network structure information is different.

With the development of deep learning, deep-link prediction based on network structure is receiving attention, such as link prediction combined with deep learning and random walks [13,14,15,16,17]. First, these methods extract structural network features on the basis of random walks to capture latent information in bipartite networks. Then, on the basis of the deep-learning framework, the results of random walks are trained to obtain a deep-link prediction model for between target nodes. The strategy based on deep matrix factorization transforms the link prediction problem into a matrix completion problem. This strategy stores the structural information of the bipartite network on the basis of the adjacency matrix and realizes the completion of the matrix by learning explicit scoring and implicit feedback in the matrix through the deep neural network [18,19]. In fact, the network structure contains the feature information of links between nodes, and the idea is to automatically learn these features from the network structure. Link prediction based on heterogeneous graph neural networks can automatically learn the structural features in the network and realize link prediction between different types of nodes [20,21,22,23].

Through the above analysis, existing link prediction methods are mainly based on the global structure of a bipartite network, relying on structural and feature information between nodes for analysis, and they lack more refined local structural analysis. To tackle this problem, this paper proposes deep-link prediction (DLP) based on the local structure of bipartite networks to leverage the local structural information of the network to enhance prediction performance. First, the local structure between target nodes is extracted, the representation learning of the local structure is then performed on the basis of the graph neural network, and deep-link prediction is lastly realized on the basis of local structure features. The main contributions in our paper are as follows:

1. The DLP method is proposed on the basis of the local structure of the bipartite network, demonstrating the rationality of the local structure in link prediction.
2. A local structure extraction algorithm was designed to effectively extract local structures between target nodes of the bipartitie network and provide local structural information for link prediction.
3. Experimental results on five datasets demonstrate the superiority of DLP over state-of-the-art link prediction methods.

## 2. Related Work

### 2.1. Link Prediction of Bipartite Networks Based on Similarity Structure

The link prediction strategy based on structural similarity mainly measures a similarity score according to the structural information between target node pairs and judges the possibility of a link between target nodes on the basis of this score. This link prediction strategy mainly includes similarity indices based on local information, paths, and random walks. The similarity index based on local information mainly realizes link prediction on the basis of common neighbors between target nodes [24,25]. Wang demonstrated the excellent link prediction performance of the CN method in biological networks. A bipartite network was constructed on the basis of DNA-binding protein and drug interaction datasets, and successfully predicted novel DNA-binding protein targets for drugs [26]. The path-based similarity index is mainly based on connected paths between target nodes as the basis for link prediction, and it can calculate local and global structural similarity between target nodes according to path length. Lu presented a local path index to estimate the likelihood of the existence of a link between two nodes and collect the data of six real networks. Extensive numerical simulations on both modeled and real networks demonstrated the high effectiveness and efficiency of the local path index compared with the common neighbors and the Katz index [27]. The similarity index based on random walks is mainly based on the relationship transfer of the network structure to analyze the possibility of a link between target nodes and realize link prediction between nodes according to the probability of the starting node walking to the target node [28]. Fouss proposed a new perspective to describe similarity between database elements with the property of it increasing with the number of paths connecting these elements. Experimental results on the MovieLens database showed that this method performs well compared to other methods [29].

### 2.2. Link Prediction of Bipartite Network Based on Machine Learning

The essence of recommendation is to predict the relationship between users and items, which is actually a variant of link prediction. The user–item recommendation system based on machine learning mainly trains the prediction model according to the feature information between target nodes to realize a recommendation [30,31]. The user–item network contains not only the feature information of users and items, but also corresponding interaction relationships. User and item representations are abstracted through multilayer perceptron (MLP), and annotated according to the interaction relationship [32,33]. The training model is obtained on the basis of features and labels. When user and item feature information is input into the model, the model produces prediction results, thereby realizing corresponding link prediction. The DSPR model extracts recommendation-oriented representations for social tags, which solves the problem of uncontrolled vocabulary and achieves superior personalized recommendations. This work also proposes to use negative sampling to greatly reduce the training time of the system and ensure good scalability in practice. Experiments based on several datasets collected from the Delicious bookmarking system showed that DSPR significantly outperformed the state-of-the-art baselines in personalized recommendations in terms of all selected metrics. In addition, when node feature information is missing, a machine-learning model can also achieve link prediction by learning the structural information between target nodes [34,35]. Matrix decomposition based on deep learning performs matrix completion by learning hidden features in the adjacency matrix to realize link prediction between corresponding nodes [36]. This work proposed a new method called deep matrix factorization (DMF) for complete matrices. DMF is able to recover missing entries of nonlinear data extracted from nonlinear latent variable models. Experimental results on toy matrix completion, image inpainting, and collaborative filtering tasks validated that DMF outperformed state-of-the-art methods for linear and nonlinear matrix completion.

### 2.3. Link Prediction Based on Heterogeneous Graph Neural Networks

The link prediction of heterogeneous graph neural networks is mainly based on the message passing framework [37,38]. It utilizes characteristics of the network structure to realize interdomain message passing and intradomain alignment towards information fusion over domains, and combines continuously updated features to realize link prediction between nodes. For example, the GCMC model achieves node embedding based on the graph convolution of the user–item network, and predicts edges on the basis of matrix factorization techniques [39]. This work proposes a new method to solve the problem of bipartite edge prediction that uses a multihop neural network structure to effectively enrich model expressiveness, and uses first-order Chebyshev approximation to greatly reduce the complexity of training time. Experimental results on benchmark datasets of collaborative filtering, citation network analysis, course prerequisite prediction, and drug–target interaction prediction showed that our method consistently outperforms several state-of-the-art methods in most cases. The THGNN model mainly applies an alternating two-step aggregation mechanism, including intra-meta-path decomposition and inter-meta-path mergence, which can distinctively aggregate rich heterogeneous information according to inferential topic-aware factors and preserve hierarchical semantics for learning multifacet node representations for link prediction [40]. The THGNN model consistently performed better than all baselines on three datasets (DBLP, YELP, and Amazon). Compared to the state-of-the-art performance of the baseline, THGNN achieved improvements in terms of both AUC and AP, which indicated the effectiveness of delicate designs for factorizing multifacet topic-aware semantics in THGNN. Type-aware anchor link prediction across heterogeneous network (TALP) simultaneously considers the effect of type and fusion information on user node alignment from local and global perspectives, solves the problem of network embedding and type-aware alignment, and performs link prediction on the basis of node embedding information [41]. TALP is evaluated through two pairs of real-word heterogeneous networks, Aminer-Mag and Twitter-Foursquare. Results demonstrate that this method outperformed state-of-the-art approaches that predict anchor links by only considering pairwise similarity between fusion vectors.

Although the three works above can achieve link prediction, the link prediction of bipartite networks based on a similarity structure only considers neighbors and paths of nodes in a bipartite network, and cannot mine links between nodes from a deep perspective. This method is the simplest link prediction framework, and its prediction performance is usually not excellent. The link prediction of bipartite networks based on machine learning considers the feature information of nodes and introduces structural information between nodes, which can effectively realize link prediction. However, this method can only extract part of the structural information of the network and cannot effectively extract information from the network structure for link prediction. Link prediction based on heterogeneous graph neural networks can combine node features and network structure to predict links between nodes, and can fully mine the structural information of bipartite networks. However, this method takes the entire bipartite network as the research object and cannot analyze the influence of the local structure on link prediction. Therefore, we propose deep-link prediction based on the local structure of the bipartite network that analyzes the possibility of links between nodes from the perspective of the local structure and combines deep learning for link prediction. Experimental results on five datasets showed that our proposed method can effectively achieve link prediction. At the same time, the effectiveness of the local structure in link prediction is demonstrated.

## 3. DLP Method

**Figure 2:**
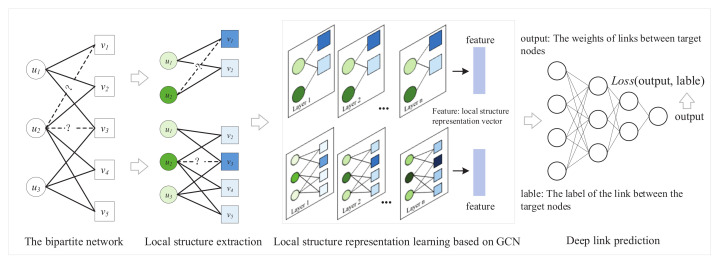
Framework of DLP method.

The specific framework of DLP is shown in Figure 2. The method can extract the local structure around the target nodes and obtain the possibility of links through local structural features. First, DLP extracts the local structure between target nodes. Then, the local structure and node features are input into the GCN model to obtain the feature representation of the local structure. Lastly, a deep-link prediction model is trained on the basis of feature representation and the link relationship between target nodes to achieve link prediction.

### 3.1. Local Structure Extraction

DLP extracts the local structure between target nodes, and on the basis of the local structure, it is used as the basis to examine whether there is a link between target nodes *u* and *v*. We designed a local structure extraction algorithm of bipartite networks that can generate all *k*-hop paths between target nodes *u* and *v* in a bipartite network and realize local structure extraction. The algorithm generates paths between nodes on the basis of depth-first-search, and the specific process is shown in Algorithm 1. First, target nodes *u* and *v*, bipartite network *G*, and path hops *k* are inputs. Then, all *k*-hop paths of node *u* are obtained on the basis of input path length *k*. If node *u* reaches node *v* through *k* hops, paths are preserved, and nodes in the paths are used to induce a local structure from bipartite network *G*, which contains the path where nodes *u* and *v* are directly connected by *k* hops. Lastly, an edge between *u* and *v* in the local structure is used as the label of the local structure.
**Algorithm 1** Local Structure Extraction**Input:** Target nodes *u* and *v*, bipartite network *G*, path hops *k***Output:** Local *k*-hop structure $G^{k}$(*u*, *v*) between node pair (*u*, *v*)1: Node = {}2: Path = {*u*: *u*∼$u_{k}$}; path is the set of *k*-hop paths of *u*, and $u_{k}$ is the end point of *k*-hop path3: **for**
*i* in Path
**do**4:  **if**
$u_{k}$ == *v*
**then**5:    node = {set of nodes in path *i* }6:    Node = Node ∪ node7: Let $G_{k}$(*u*, *v*) be the induced subgraph from *G* using vertices Node8: Remove the edge between *u* and *v* in local structure $G^{k}$(*u*, *v*), and use the edge as the label of the local structure9: **return**
$G^{k}$(*u*, *v*), label

### 3.2. Node Feature Labeling

The local structure between target nodes is obtained by Algorithm 1. The features of nodes are usually not available, so the feature labeling of different nodes is required. The purposes of node labeling are as follows:

(1) Using different labels to mark nodes’ different roles in the local structure. There are two types of nodes in a bipartite network. Distinguishing types of nodes can assist in fully understanding roles (user or item) in the local structure and effectively identify the target node.
(2) Distinguishing positions of nodes in the local structure. Nodes with different relative positions to the target node have different structural importance to the link. Distinguishing the position information of nodes can effectively extract the semantic information of target nodes in local structures.

On the basis of the above criteria, different nodes were labelled in the local structure. First, different types of nodes are labeled. There are two types of nodes, *U* and *V*, in bipartite networks. In this paper, the *U*- and *V*-type nodes were labeled as 0 and 1, respectively, to effectively distinguish between different types of nodes. Then, labels of other nodes were determined according to which hop of the target node in the local structure. If the target node went through *h* hops to the other node, we labeled it *h*, which could distinguish the node’s position in the local structure. Lastly, one-hot encoding was performed on the labels of the node, and the final feature representation of the node was obtained by splicing.

### 3.3. Local Structure Representation

The local structure and node features were used as the input of GCN for the feature representation learning of the local structure. First, message passing is performed on the basis of the adjacency matrix of the local structure and node features to realize the feature update of each node. We used the graph convolutional neural network framework to update nodes, and the formula is as follows:

(1) $$h_{i}^{l+1}=\sigma \left(h_{i}^{l}+\sum_{j\in N_{r}\left(i\right)}W_{j}^{l}h_{j}^{l}\right)$$

where $h_{i}^{l}$ represents the feature vector of node *i* in layer *l*, $N_{r}\left(i\right)$ represents the neighbors of node *i*, $h_{j}^{l}$ represents the feature vector of node *j* in layer *l*, $W_{j}^{l}$ is a learnable parameter, and σ is the ReLU activation function. Lastly, feature $h_{i}^{l+1}$ of the *l*+1 layer of node *i* is obtained.

Then, a representation method of the local structure was designed to realize feature representation. The features of the nodes in the local structure were averaged here as the feature representation of the final local structure, which not only contained the feature information of all nodes, but also ensured that the dimensions of local-structure and node features were the same.

(2) $$H_{ls}=Mean\left(h_{1},h_{2},...,h_{n+m}\right)$$

where Mean represents the mean function, $h_{1}$, $h_{2}$, …, $h_{n+m}$ represent the node feature vector in the local structure, *n* and *m* represent the number of two types of nodes, and $H_{ls}$ represents the final feature representation of the local structure.

### 3.4. Link Prediction

After local structure representation is obtained, the link score between target nodes is output on the basis of multilayer perceptron (MLP):

(3) $$r_{pre}=\sigma \left(WH_{ls}\right)$$

where W is a parameter of the MLP that maps local structural representation $H_{ls}$ to a scalar rating $r_{pre}$, and σ is the activation function. The loss function is *mean squared error* (MSE), which is used to minimize the difference between predictions and ground-truth ratings:

(4) $$Loss=\frac{1}{mn}\sum_{i=0}^{n}\sum_{j=0}^{m}{\left(R_{ij}-R_{ij}^{*}\right)}^{2}$$

where we used $R_{ij}$ and $R_{ij}^{*}$ to denote the true rating and predicted rating of (*i*, *j*), respectively.

## 4. Experiments

Our experiments were carried out on a workstation equipped with Intel(R) Xeon(R) Gold 6132 CPU @ 2.60 GHz, NVIDIA Geforce GTX 1080Ti GPU and 192 GB RAM. The modules of DLP in the experiments were implemented with Python 3.8.3 with Pytorch 1.11.0. The DLP model adopted the SGD optimizer with parameters lr = $1e^{-2}$, momentum = 0.9, weight _ decay = $1e^{-5}$. Model training parameters: epoch = 80, batch _ size = 5. The main source codes are provided here: https://github.com/lvhehe/ISAT-Laboratory, accessed on 29 March 2022.

### 4.1. Datasets

The five datasets used in this paper were collected from the KONECT Project [42]: Corporate Club Memberships Dataset (CCMD), Corporate Leaderships Dataset (CLD), American Revolution Dataset (ARD), Crime Dataset (CD), and Unicode Languages Dataset (ULD). Dataset statistics are summarized in Table 1.

### 4.2. Method Comparison

DMF [43]: This method is based on a multilayer neural network that characterizes users and items into low-dimensional features, and synthesizes a recommendation matrix via potential low-dimensional features to achieve item recommendations.

VAE [44]: This method is based on autoencoder (AE) in which the encoder represents potential features on the basis of probability distribution and reconstructs the input on the basis of the decoder.

DAE [45]: This method is based on the backpropagation algorithm, which compresses the input into a low-dimensional feature space, then reconstructs the output, and represents nodes through the low-dimensional feature space.

IGMC [46]: This method trains a graph neural network (GNN) on the basis of 1-hop subgraphs around pairs (user, item) generated from the rating matrix, and maps these subgraphs to their corresponding ratings, enabling user rating predictions for items.

### 4.3. Evaluation Indicators

To evaluate the performance of the method, we randomly removed 20% of the existing links from each dataset as positive testing data. On the basis of standard learned link prediction, the same number of nonexistent links (unconnected node pairs) were sampled as negative testing data. The remaining 80% of existing links and the same number of additionally sampled nonexistent links were used to construct training data. *Mean absolute error* (*MAE*) and *root mean square error* (*RMSE*) were used for performance evaluation.

(5) $$\begin{array}{c} MAE=\frac{1}{l}\sum_{(i,j)\in l}\left|r_{ij}-r_{ij}^{*}\right| \end{array}$$

(6) $$RMSE=\sqrt{\frac{\sum_{(i,j)\in l}{\left(r_{ij}-r_{ij}^{*}\right)}^{2}}{l}}$$

where $r_{ij}$ is the true value between nodes *i* and *j*, $r_{ij}^{*}$ is the predicted value of the DLP method, and *l* is the sample size of the experimental data.

### 4.4. Result

#### 4.4.1. Local Structural Analysis

In this paper, the extraction of the local structure is mainly based on the *k*-hop path between target nodes; therefore, the influence of local structural depth on method performance is studied. Results are shown in Table 2; when local structural depth *k* was 1, DLP achieved the best performance of all five datasets in terms of RMSE and MAE. In addition, the performance of DLP on CCMD, CLD, and ULD was first worsened and improved when *k* increased from 1 to 3. This shows that, when *k* increases, the method captures deeper structural information among target nodes. However, on ARD and CD, the performance of the method gradually worsened as depth *k* increased. This shows that, when *k* increases, information between target nodes captured by the local structure tends to be similar, and the method cannot accurately distinguish structural differences between target nodes.

#### 4.4.2. Performance Comparison

The performance of DLP was compared with that of the aforementioned state-of-the-art methods, and results are shown in Table 3. The following conclusions were drawn:

(1) In terms of RMSE and MAE, DLP achieved the best performance on the five datasets, indicating that DLP could effectively predict links existing between nodes.
(2) On the five datasets, the overall reduction in MAE showed that DLP could reduce the error between predicted and actual values, and achieved effective prediction of edge weights between nodes.
(3) The overall reduction in RMSE of the DLP method on the five datasets was 0.498, 0.619, 0.285, 0.605 and 0.601. DLP achieved the smallest RMSE on all datasets, indicating that the prediction results of the method were stable.

#### 4.4.3. Influence of Link Sparsity

**Figure 3:**
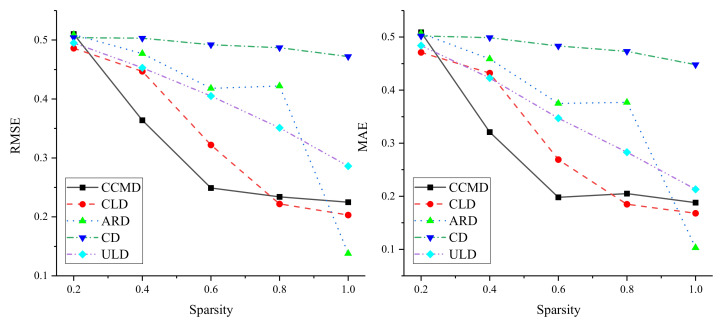
Results of sparsity analysis.

In order to verify the applicability of the method, sparsity was experimentally analyzed on five different datasets. The influence of link sparsity on DLP performance was investigated by randomly retaining linked 0.2, 0.4, 0.6, 0.8, and 1.0 in the original dataset. Results are shown in Figure 3. On the five datasets, experiments showed that the performance of DLP improved with increasing sparsity, indicating that the method relies on dense bipartite networks. At the same time, on CCMD and CLD, RMSE and MAE decrease rapidly on the basis of the increase in links. When sparsity reached 0.8, as it increased, although the performance of the method improved, RMSE and MAE gently decreased. On CD and ULD, the RMSE and MAE of DLP showed a steadily decreasing trend, which indicated that the sparsity of these two datasets more smoothly impacted DLP performance. In addition, on ARD, DLP performance showed fluctuating improvement based on the increase in links. When sparsity increased from 0.8 to 1.0, the performance of DLP suddenly improved, which indicated that DLP is more affected by sparsity on ARD, that is, the denser the edges in the bipartite network are, the better the performance of DLP is.

#### 4.4.4. Model Analysis

DLP can extract the local structure of a bipartite network for analysis, analyze the local relationship between nodes, and study the importance of the local structure in link prediction. Experiments showed that DLP outperformed methods based on a global structure, proving the effectiveness of the local structure in link prediction. However, analysis of link sparseness showed that DLP can achieve excellent link prediction performance on dense bipartite networks, but relatively poor performance on sparse bipartite networks. This is understandable because the extraction of a local structure depends on the link relationship in the network. When the link relationship between nodes is sparse, the local structure is simple, and thefeatures of the local structure cannot be effectively extracted. This method is only based on artificially defined features and cannot effectively express features of nodes. Therefore, future research will collect node feature information as supplementary network information to achieve more effective link prediction.

## 5. Conclusions

Novel link-prediction method DLP was proposed that could mine feature information on the basis of the local structure between target nodes of bipartite networks. The following conclusions were drawn:

(1) The effectiveness of the local structure in link prediction was confirmed, and experiments on five datasets showed that, when the local structure is smaller, the performance of link prediction is better.
(2) Compared with existing state-of-the-art methods, DLP showed excellent performance on RMSE and MAE, indicating that this method could achieve effective prediction of link presence and link weight between nodes.
(3) In addition, the performance of the method was improved on the basis of the increase in the sparsity of the dataset, indicating that the method is more suitable for dealing with dense bipartite networks.

## Figures and Tables

**Table 1:** Statistics of five commonly used link prediction datasets.

Dataset	*U*	*V*	Interaction	Description
CCMD	25	15	95	Membership information of clubs and boards
CLD	20	24	99	Person and company leadership information
ARD	136	5	160	Membership between persons and organizations
CD	829	551	1476	Relationship between suspect and crime
ULD	254	614	1255	Spoken relationship between country and language

**Table 2:** Relationship between DLP performance and local structural depth.

Dataset	*k* = 1		*k* = 2		*k* = 3	
	*RMSE*	*MAE*	*RMSE*	*MAE*	*RMSE*	*MAE*
CCMD	0.225	0.188	0.549	0.494	0.255	0.199
CLD	0.203	0.168	0.506	0.485	0.297	0.283
ARD	0.138	0.103	0.175	0.124	0.208	0.187
CD	0.472	0.448	0.480	0.458	0.495	0.483
ULD	0.286	0.213	0.587	0.564	0.322	0.307

**Table 3:** Performance comparison of DLP and various state-of-the-art methods.

Method	CCMD		CLD		ARD		CD		ULD	
	*RMSE*	*MAE*	*RMSE*	*MAE*	*RMSE*	*MAE*	*RMSE*	*MAE*	*RMSE*	*MAE*
DMF	0.483	0.437	0.484	0.472	0.468	0.407	0.497	0.452	0.434	0.366
VAE	0.519	0.451	0.479	0.400	0.506	0.477	0.495	0.449	0.433	0.353
DAE	1.432	1.043	1.818	1.290	0.404	0.189	2.802	1.296	2.181	1.030
IGMC	0.458	0.451	0.508	0.503	0.314	0.295	0.512	0.498	0.500	0.478
DLP	0.225	0.188	0.203	0.168	0.138	0.103	0.472	0.448	0.286	0.213

## Data Availability

Not applicable.
